# Serum Zinc Level in Children Presenting with Febrile Seizures

**Published:** 2013

**Authors:** Muhammad Waqar Rabbani, Ibad Ali, Hafiz Zahid Latif, Abdul Basit, Muhammad Ali Rabbani

**Affiliations:** 1 **Dr. Muhammad Waqar Rabbani, DCH, FCPS, Department of Pediatric Medicine, The Children Hospital & The Institute of Child Health, Multan, Pakistan.**; 2 **Dr. Ibad Ali, DCH, FCPS, Department of Pediatric Medicine, The Children Hospital & The Institute of Child Health, Multan, Pakistan.**; 3 **Dr. Hafiz Zahid Latif, MBBS, Department of Pediatric Medicine, The Children Hospital & The Institute of Child Health, Multan, Pakistan.**; 4 **Dr. Abdul Basit, MBBS, Department of Pediatric Medicine, The Children Hospital & The Institute of Child Health, Multan, Pakistan.**; 5 **Muhammad Ali Rabbani, Undergraduate MBBS student, Nishtar Medical College, Multan, Pakistan.**

**Keywords:** Febrile Seizure, Convulsion, Serum zinc level, Epilepsy

## Abstract

***Objective:*** To determine the frequency of low serum zinc level in children presenting with febrile seizures at The Children’s Hospital and the Institute of Child Health (CH/ICH) Multan.

***Methods:*** This is an observational cross sectional study conducted at the Department of Pediatric Medicine, The Children’s Hospital and the Institute of Child Health, Multan from September 2010 to March 2011. Children (6 months to 6 years of age) presenting with febrile seizures who satisfied inclusion and exclusion criteria were enrolled for the study. Cause of fever was determined after detailed history, physical examination and relevant investigations. Four milliliters centrifuged blood sample was preserved in acid washed test tube. Separated serum was used to measure serum zinc level by employing Randox kit on auto-analyzer in all cases. The outcome variable (serum zinc level) was recorded on a predesigned proforma.

***Results:*** Out of 100 enrolled children, there were 66 (66%) male with male to female ratio of 1:0.52. Mean age of the children was 23.97±14.45 months. Upper respiratory tract infection was the most frequent cause of fever apparent in 24 children (24%) followed by tonsillitis 17 (17%), pneumonia 16 (16%), urinary tract infection 16 (16%), otitis media 15 (15%), and bronchiolitis 12 (12%). Frequency of low serum zinc level was 26% in children with febrile seizures.

***Conclusion:*** Zinc deficiency could be a potential risk factor for febrile seizure in children.

## INTRODUCTION

A seizure is a paroxysmal event caused by abnormal electrical discharge inside the brain.^1^ Febrile convulsion; twice as common in boys as in girls, is one of the most common type of seizure occurring in children between 5 months and 6 years of age, accounting for 30% of all seizures in children.^2^^,^^3^ This is an age dependent response of the immature brain to fever in children,^4^ who do not have an intracranial infection, metabolic disturbance, or history of afebrile seizures.^5^ Eighty to eighty five percent febrile seizures occur between 6 months and 3 years of age, with peak incidence at 18 months.^6^^,^^7^ Children with a simple febrile seizure has potential for recurrence and 2-7% of children may develop epilepsy by adolescence.^8^^,^^9^ Contrary to simple febrile seizure, complex febrile seizures are prolonged (>15 minutes), focal and occur more than once in 24 hours.^10^

Zinc is an important micronutrient that plays a significant role in growth and development, immune system response, enzymatic activity of different organs, proteins and cellular metabolism, neurological functions, nerve impulse transmission and hormone release.^11^^,^^12^ The possible role of zinc deficiency in provoking febrile seizures has been reported in different studies.^13^^,^^14^ Zinc stimulates the activity of pyridoxal kinase, the enzyme that modulates GABA level, a major inhibitory neurotransmitter.^15^ It also modifies the affinity of neurotransmitters and thus prevents the excitatory neuronal discharge.^2^ In addition, zinc significantly reduces the severity of illness and the duration of fever in children with pneumonia and diarrhea by the activation of immune enhancing T-cells.^11^^,^^16^

Earlier no such study has been done in this region to see the association between low serum zinc level and febrile fits. This study will provide a base line data and help in the formulation of guidelines for zinc supplementation as a part of management of febrile fits.

## METHODS

This study was carried out from September 2010 to March 2011 at the department of Pediatric Medicine, the CH/ICH Multan, Pakistan. Patients, 6 months to 6 years of age presenting in Emergency department with fever (≥ 38 ºC) and history of fits within last 6 hours, having normal cerebrospinal fluid examination and normal; serum glucose, sodium, potassium, calcium and magnesium levels were included. The patients who had any evidence of central nervous system infection, epilepsy, metabolic seizures or those who are already on zinc therapy for any other ailment like malnutrition, diarrhea, pneumonia or acrodermatitis enteropathica were excluded.

This cross sectional study was performed after permission from Institutional Ethical Committee. All the details of the study were explained to the parents/guardians and informed consent was taken. Cause of fever was determined by detailed history, complete physical examination and relevant investigations (complete blood count, complete urine examination, malarial parasite, and typhidot test). CSF examination was done in all patients younger than 1.5 years of age and selective older patients who had any clinical suspicion of central nervous system infection. Serum zinc level was assessed in all patients within 6 hours of febrile seizure. Four milliliters centrifuged blood sample was preserved in acid washed test tube. Separated serum was used to measure serum zinc level by employing Randox kit UK on auto-analyzer in the chemical pathology laboratory of the hospital. Normal values of serum zinc level were defined from 70–120 μg/dl in pediatric age group (5 months to 6 years).

The data was analyzed using computer program SPSS-10. Descriptive statistics were applied. Quantitative variable like age was analyzed by mean and standard deviation. Qualitative variables like gender and serum zinc level which is an outcome variable (normal/abnormal) were analyzed by taking frequencies and percentages.

## RESULTS

Hundred children (6 months to 6 years of age) with febrile seizure were included in the study. Mean age of the children was 23.97±14.45 months. Frequency of low serum zinc level was 26% in children with febrile seizures. Sixty three (63%) children were between 6 months to 3 years of age while rest of the 37(37%) were between >3 years to 6 years of age. Low serum zinc level in different age groups is given in Table-I. There were 66 (66%) male children with a male to female ratio of 1:0.52, frequency of low serum zinc level with reference to male and female patients has been described in Table-II.

Upper respiratory tract infection was the most frequent cause of fever evident in 24 children (24%) followed by tonsillitis 17 (17%), pneumonia 16 (16%), urinary tract infection 16 (16%), otitis media 15 (15%), and bronchiolitis 12 (12%) in children having febrile seizures. Rate of low serum zinc level in various causes of fever is mentioned in Table-III.

**Table-I:** Low Serum Zinc Level in Febrile Seizure Patients in Relation to Age Groups

***Age in years***	***No. of Patients***	***No. of Patients with*** ***Low Serum Zinc***	***Percentage (%)***
**0.6 – 3**	**63**	**15**	**23.8**
**>3 – 6**	**37**	**11**	**29.7**
**Total**	**100**	**26**	

**Key: Age is rounded to the nearest year**

**Table-II:** Low Serum Zinc Level in Febrile Seizure Patients in Relation to Gender

***Sex***	***Total No. of*** ***Patients***	***No. of Patients with*** ***Low Serum Zinc***	***Percentage (%)***
**Male**	**66**	**19**	**28.8**
**Female**	**34**	**7**	**20.6**
**Total**	**100**	**26**	

**Table-III:** Low Serum Zinc Level in Febrile Seizure Patients with Relation to Causes of Fever

***Causes***	***Total No. of Patients***	***No. of Patients with Low Serum Zinc***	***Percentage (%)***
**Upper respiratory tract infection**	**24**	**6**	**25.0**
**Tonsillitis**	**17**	**5**	**29.4**
**Pneumonia**	**16**	**5**	**31.3**
**Urinary tract infection**	**16**	**5**	**31.3**
**Otitis media**	**15**	**3**	**20.0**
**Bronchiolitis**	**12**	**2**	**16.7**
**Total**	**100**	**26**	

## DISCUSSION

Febrile seizure is a commonly occurring problem in young children. Although its pathogenesis is debatable, studies have revealed that the genetic factors, family background, immunologic disorders, iron deficiency and zinc deficiency may play a role in febrile seizure. The infection state exhibits non-specific host responses, including immune responses such as changes in the concentrations of certain plasma proteins, cytokines (tumor necrosis factor, interleukin-1, interleukin-6) and interferon which may result in reduction of serum zinc level. Hypozincemia has been suggested as a possible change during the rising phase of body temperature in febrile patients.^17^

This study was conducted at the CH/ICH Multan. Age of presentation in majority of children (63%) was 6 months to 3 years. Frequency of low serum zinc level was found in 26% children with febrile seizures in this study, which is comparable with several international studies in children with febrile seizure. Mollah MA et al^14^ in 2008 published a study comparing serum and CSF Zinc levels of febrile seizure children to their matched non-seizure febrile peers. Mean Zn concentration in both serum and CSF was less in febrile seizure children than in their matched non-seizure febrile peers (p < 0.001). Kumar L et al^18^ in a recent case control study found that mean serum zinc level was significantly lower in cases as compared to control (p<0.05) in children having febrile seizure. Ganesh R et al^13^ compared serum zinc levels in 38 cases of simple febrile seizure with 38 age matched controls with statistically significant results (p<.001). Amiri M et al,^19^ Modarresi MR et al,^20^ Hydarian F et al,^21^ Lee J and Kim JH,^22^ and Talebian A et al,^23^ also gave similar results which are comparable with our study. However Garty BZ et al^24^ had their findings which did not support the hypothesis that febrile convulsions are related to the reduced zinc concentration.

One limitation of our study is the lack of a control group. Furthermore, it was done in small number of patients. As the base line zinc level was lacking in these febrile seizure patients, which prevented us from reaching a definite conclusion whether infectious diseases were responsible for low zinc levels or there was pre-existing hypozincemia. More prospectively designed, multi center studies involving larger sample sizes are needed to answer these questions.

## CONCLUSION

In conclusion, this study revealed that the low serum zinc levels (26%) in our study are fairly sufficient to support the hypothesis that Zinc deficiency could be a potential risk factor for febrile seizure in children.

## Authors Contribution:

*Rabbani MW, Ali I, Latif HZ and Basit A:* Conceived, designed, manuscript writing, editing and review of manuscript. *Rabbani MA:* Editing and review of manuscript.

## References

[1] Margaretha L, Masloman N (2010). Correlation between serum zinc level and simple febrile seizure in children. Paediatr Indones.

[2] Ehsanipour F, Talebi-Taher M, Harandi NV, Kani K (2009). Serum zinc level in children with febrile convulsion and its comparison with that of control group. Iran J Pediatr.

[3] Vestergaard M, Obel C, Henriksen TB, Christensen J, Madsen KM, Ostergaard JR (2006). The Danish National Hospital Register is a valuable study base for epidemiologic research in febrile seizures. J Clin Epidemiol.

[4] Jensen FE, Sanchez RM, Baram TZ, Shinnar S (2002). Why does the developing brain demonstrate heightened susceptibility to febrile and other provoked seizures. Febrile seizures.

[5] Steering Committee on Quality Improvement, Management, Subcommittee on Febrile Seizures (2008). Febrile seizures: clinical practice guideline for the long-term management of the child with simple febrile seizures. Pediatrics.

[6] Shinnar S, Glauser TA (2002). Febrile seizures. J Child Neurol.

[7] Waruiru C, Appleton R (2004). Febrile seizures: an update. Arch Dis Child.

[8] Habib Z, Akram S, Ibrahim S, Hasan B (2003). Febrile seizures: factors affecting risk of recurrence in Pakistani children presenting at the Aga Khan University. Hospital J Pak Med Assoc.

[9] Elkis LC, Manreza MLG, Grossmann RM, Valerio RMF, Guilhoto LMFF (2003). Crises sintomaticas agudas. Epilepsia (infancia e adolescencia).

[10] Karande S (2007). Febrile seizures: a review for family physicians. Indian J Med Sci.

[11] Bhandari N, Bahl R, Taneja S, Strand T, Molbak K, Ulvik RJ (2002). Effect of routine zinc supplementation on pneumonia in children aged 6 months to 3 years: randomised controlled trial in an urban slum. BMJ.

[12] Mahyar A (2005). The preventive role of zinc from communicable and non-communicable diseases in children. NCD Malaysia.

[13] Ganesh R, Janakiraman L (2008). Serum zinc levels in children with simple febrile seizure. Clin Pediatr (Phila).

[14] Mollah MA, Rakshit SC, Anwar KS, Arslan MI, Saha N, Ahmed S (2008). Zinc concentration in serum and cerebrospinal fluid simultaneously decrease in children with febrile seizure: findings from a prospective study in Bangladesh. Acta Paediatr.

[15] Burhanoglu M, Tutuncuoglu S, Coker C, Tekgul H, Ozgur T (1996). Hypozincaemia in febrile convulsion. Eur J Pediatr.

[16] Raqib R, Roy SK, Rahman MJ, Azim T, Ameer SS, Chisti J (2004). Effect of zinc supplementation on immune and inflammatory responses in pediatric patients with shigellosis. Am J Clin Nutr.

[17] Ehsani F, Vahid-Harandi M, Kany K (2006). Determination of serum zinc in children affected by febrile convulsion and comparison with control group. The J Iranian Medi Sci Uni.

[18] Kumar L, Chaurasiya OS, Gupta AH (2011). Prospective study of level of serum zinc in patients of febrile seizures, idiopathic epilepsy and CNS infections. People’s J Scientific Research.

[19] Amiri M, Farzin L, Moassesi ME, Sajadi F (2010). Serum trace element levels in febrile convulsion. Biol Trace Elem Res.

[20] Modarresi MR, Shahkarami SMA, Yaghini O, Shahbi J, Mosaiiebi D, Mahmoodian T (2011). The relationship between zinc deficiency and febrile convulsion in Isfahan, Iran. Iranian J Child Neurol.

[21] Heydarian F, Ashrafzadeh F, Ghasemian A (2010). Serum Zinc level in patients with simple febrile seizure. Iranian J Child Neurol.

[22] Lee JH, Kim JH (2012). Comparison of serum zinc levels measured by inductively coupled plasma mass spectrometry in preschool children with febrile and afebrile seizures. Ann Lab Med.

[23] Talebian A, Vakili Z, Talar SA, Kazemi SM, Mousavi GA (2009). Assessment of the relation between serum zinc & magnesium levels in children with febrile convulsion. Iranian J Pathol.

[24] Garty BZ, Olomucki R, Lerman-Sagie T, Nitzan M (1995). Cerebrospinal fluid zinc concentrations in febrile convulsions. Arch Dis Child.

